# Direct puncture of the carotid artery as a bailout vascular access technique for mechanical thrombectomy in acute ischemic stroke—the revival of an old technique in a modern setting

**DOI:** 10.1007/s00234-020-02520-x

**Published:** 2020-08-15

**Authors:** Milena Miszczuk, Hans Christian Bauknecht, Justus F. Kleine, Thomas Liebig, Georg Bohner, Eberhard Siebert

**Affiliations:** 1 Institute of Neuroradiology, Charité–Universitary Medicine Berlin, Charitéplatz 1, 10117 Berlin, Germany; 2 Institute of Neuroradiology, Ludwig Maximillian University Munich, Munich, Germany

**Keywords:** Mechanical thrombectomy, Direct carotid puncture, Stroke, Large vessel occlusion

## Abstract

**Purpose:**

To describe our single-center experience of mechanical thrombectomy (MTE) via a direct carotid puncture (DCP) with regard to indication, time metrics, procedural details, as well as safety and efficacy aspects.

**Methods:**

DCP thrombectomy cases performed at our center were retrospectively identified from a prospectively maintained institutional MTE database. Various patient (age, sex, stroke cause, comorbidities), clinical (NIHSS, mRS), imaging (occlusion site, ASPECT score), procedural (indication for DCP, time from DCP to reperfusion, materials used, technical nuances), and outcome data (NIHSS, mRS) were tabulated.

**Results:**

Among 715 anterior circulation MTEs, 12 DCP-MTEs were identified and analyzed. Nine were left-sided M1 occlusions, one right-sided M1 occlusion, and two right-sided M2 occlusions. DCP was successfully carried out in 91.7%; TICI 2b/3-recanalization was achieved in 83.3% via direct lesional aspiration and/or stent-retrieval techniques. Median time from DCP to reperfusion was 23 min. Indications included futile transfemoral catheterization attempts of the cervical target vessels as well as iliac occlusive disease. Neck hematoma occurred in 2 patients, none of which required further therapy.

**Conclusion:**

MTE via DCP in these highly selected patients was reasonably safe, fast, and efficient. It thus represents a valuable technical extension of MTE, especially in patients with difficult access.

## Introduction

Mechanical thrombectomy (MTE) is now the accepted standard of care for patients with acute ischemic stroke due to large vessel occlusion (LVO) as shown by various randomized-controlled trials [1].

Many factors have impact on the stroke outcome, with the quality of MTE and the time to reperfusion being crucial. Many stroke patients suffer from generalized cardiovascular morbidity. Thus, atherosclerotic, dissecting, or occlusive disease of the access vessels (femoral arteries, iliac arteries, subclavian arteries, and aorta) has a high prevalence in this population and may impose obstacles to or even prevent safe and timely catheterization [2]. Furthermore, severe vessel elongation, kinking, and looping, as well as aortic arch remodeling secondary to chronic hypertensive and atherosclerotic disease may hinder selective catheterization and navigation in the supraaortic arteries, frequently aggravated by loss of body height secondary to vertebral compression fractures and disc degeneration with resulting tortuous vessel courses in elderly patients, especially women. Altogether, these may severely prolong selective catheterization and counteract the stability of co-axial catheter constructs necessary for successful intracranial MTE [3–5].

These procrastinating factors influence the time spent from groin puncture to thrombus retrieval and are therefore likely to also have a negative impact on patient outcome as shown in previous studies [3–5]. Direct carotid puncture (DCP) may provide an elegant solution to quickly establish vascular access and provide a stable platform for subsequent MTE. In fact, DCP was the initial standard access route in cerebral angiography before being supplanted by transfemoral, transbrachial, and more recently also transradial approaches as catheter navigability improved [6–9].

While casuistic descriptions of DCP exist for treatment of intracranial aneurysms, carotid stenosis as well as for stroke MTE, the literature on the topic is still scarce. Yet, we believe that this technique may represent a valuable bailout access technique for MTE in selected cases. Therefore, we present our series of 12 cases in which we used a DCP approach for MTE (DCP-MTE) in patients with acute stroke secondary to LVO and discuss procedural details of access implementation and revascularization strategy as well as outcome and complications.

## Patients and methods

### Patients and imaging

All patients were treated in one tertiary stroke referral center according to institutional and national thrombectomy guidelines [10]. In short, patients with LVO of the anterior circulation as diagnosed by CTA, a clinically relevant neurological deficit (NIHSS > 5) and no extensive early ischemic changes (ASPECTS > 5) were offered MTE within a 6-h time window. Patients were also treated within 24 h or even beyond, if a favorable mismatch profile on CT-perfusion was present (essentially adhering to the DEFUSE 3 and DAWN criteria) [11, 12].

From a prospectively maintained institutional database of all consecutive patients treated with MTE for acute ischemic stroke from January 2017 to March 2020, patients with an anterior circulation occlusion treated by DCP-MTE were retrospectively identified. Clinical data with the following parameters were assessed: age, neurological and functional status (NIHSS and mRS) on admission, and discharge and cardiovascular stroke risk factors. Cross-sectional imaging data, such as ASPECTS and occlusion site, and angiographic features, including indication for carotid puncture, time to DCP, MTE materials and technical nuances, procedural duration, type of anesthesia, angiographic (mTICI) and clinical outcome, number of MTE maneuvers, time from onset to reperfusion, and procedure-related complications were recorded. Furthermore, the B.A.D. score (as for bovine arch, aortic arch type, ICA dolichoarteriopathy) as indicator for unfavorable vascular anatomy was assessed, as described previously [4, 13]. The study was approved by the Charité Ethics Committee (EA2/082/15). Informed consent of individual patients or of the next of kin for the dead patients was waived for this retrospective study of anonymized data according to pertinent institutional guidelines.

### Endovascular procedure

In our institution, there are 5 experienced senior neurointerventionalists treating stroke. DCP is considered in patients with an acute anterior circulation stroke if catheterization of the target vessel cannot be achieved within approximately 30 min despite trying multiple catheterization strategies or in case of aorto-biiliac occlusion. The patient is placed in a supine position with the head rotated contralaterally to the vessel occlusion. After standard sterile prepping and draping, local anesthetic with 1% lidocaine is administered to the soft tissues. The carotid puncture is performed under sonographic guidance, as proximal as possible with a 5F micropuncture set (Cook Medical Europe, Limerick, Ireland). After insertion of the micropuncture needle, a road map is performed in order to guide the insertion of the short introducer wire or a 0.035″ hydrophilic guidewire (Terumo, Tokyo, Japan) if the distal anatomy is very tortuous or undue resistance is encountered with the introducer wire. Care is taken not to push the wire beyond the ascending petrous segment. A standard short 6F vascular sheath is inserted with the tip reaching into the proximal to middle aspect of the internal carotid artery, depending on local anatomy. Alternatively, a 90-cm 6F shuttle sheath (Destination, Terumo, Tokyo, Japan) was used in conjunction with a 5-Fr aspiration catheter (SOFIA, MicroVention, Aliso Viejo, CA, USA) that is introduced with the aid of a regular guidewire in order to be able to pass the membraneous valve of the short sheath or via a flow switch Y-connector. Care was taken to fix the sheath to the skin by either suturing or using adhesive draping in order to prevent kick-back in case of forward push of the intermediate or microcatheter. MTE is then performed in a regular manner either with a direct aspiration first pass technique (ADAPT), wireless ADAPT (wADAPT)), or with stent-retriever supported technique combined with distal aspiration. Vessel closure is achieved either by careful and controlled manual compression with the ultrasound transducer or by off-label use of the AngioSeal (St. Jude Medical Inc. Minnetonka, MN, USA) vascular closure device.

## Results

### Patients

Between January 2017 and March 2020, we performed 715 MTEs for acute anterior circulation ischemic strokes at our institution. Out of these, 708 were primarily approached by a transfemoral access, with 679 being successful concerning establishment of a stable cervical target vessel position and 29 being unsuccessful in this respect. Out of the latter, 11 procedures were aborted and 10 cases were converted to a secondary DCP. Four cases each were converted to a transbrachial and transradial approach. In 2 cases, primary DCP was performed because of aorto-biiliac artery occlusion. In that time, a total of 6 transbrachial approaches (3 for vertebrobasilar and 3 for right anterior circulation MTE) and, after establishment of the transradial route in August 2019 in our department, 7 transradial approaches (all for vertebrobasilar MTE) have been performed. The study cohort presented here thus comprises a total of 12 DCP-MTE cases (1.66 % of all MTEs). Demographic, clinical, and imaging data are detailed in Tables 1 and 2. In short, the median age of the DCP-MTE patient group was 85.5 years (range 74–94); 8 patients were female (66.7%). The median NIHSS at presentation was 16 (range 12–26); the median pre-stroke mRS was 3.5 (2–5). Most patients had hypertension (83.3%) or showed atrial fibrillation and hyperlipidemia (66.6% and 58.3%, respectively, see Table 2). Cardiac embolism represented the most common cause of stroke (*n* = 8). Eight patients were treated under general anesthesia and 4 under conscious sedation.

**Table 1:** Patients, time metrics, procedural details, and outcome. *ASPECTS*, Alberta Stroke Program Early CT Score; *B.A.D*. *score*, bovine arch, aortic arch type, ICA dolichoarteriopathy score (Smelling et al. 2018); *CCA*, common carotid artery; *CS*, conscious sedation; *DCP*, direct carotid puncture; *GA*, general anesthesia; *HT1*, hemorrhagic transformation type 1 (ECASS criteria); *ICA*, internal carotid artery; *ICH*, intracerebral hematoma; *mTICI*, modified treatment in cerebral ischemia score; *NIHSS*, National Institutes of Health Stroke Scale; *PH2*, parenchymal hematoma type II; *SAH*, subarachnoid hematoma; *at discharge

	Patient 1	Patient 2	Patient 3	Patient 4	Patient 5	Patient 6	Patient 7	Patient 8	Patient 9	Patient 10	Patient 11	Patient 12
Age	94	87	92	86	85	88	79	81	81	74	92	76
Sex	Female	Female	Female	Female	Female	Female	Male	Female	Female	Male	Male	Male
NIHSS pre	21	13	19	14	17	16	12	16	26	14	9	19
NIHSS post*	19	-	-	12	-	-	11	2	-	-	1	15
ASPECTS	10	9	8	8	8	8	9	8	8	9	10	9
mRS pre	4	3	4	3	2	2	5	2	5	5	3	1
mRS post*	4	6	6	3	6	6	4	4	6	6	2	4
Occlusion site	Left M1	Left M1	Left M1	Left M1	Left M1	Right M1	Left M1	Left M1	Left M1	Left M1	Right M2	Right M2
Anesthesia	CS	GA	CS	GA	GA	GA	GA	GA	GA	CS	CS	CS ➔ GA
Reason DCP	Type 3 aortic arch, bicarotid trunk, acute CCA take-off angle, goiter with dorso-lateral CCA shift	Bifemoral aortoiliac occlusive disease	Type 3 aortic arch, acute CCA take-off angle, goiter with dorso-lateral CCA shift	Type 3 aortic arch, acute CCA take-off angle, proximal CCA kinking	Bifemoral aortoiliac occlusive disease	Type 2 aortic arch, severe CCA tortuosity	Bifemoral aortoiliac occlusive disease	Type 2 aortic arch, aortic arch elongation, severe scoliosis	Type 3 aortic arch, bovine arch, severe tortuosity of iliac arteries and aorta	Bifemoral aortoiliac occlusive disease	Type 3 aortic arch, severe ICA tortuosity	Type 2 aortic arch, severe ICA tortuosity
B.A.D. score	3	2	3	3	1	2	1	2	4	0	3	2
Time from groin puncture to DCP	30 min	-	35 min	20 min	-	30 min	15 min	120 min	45 min	31 min	20 min	64 min
DCP until reperfusion	19 min	73 min	18 min	50 min	13 min	27 min	65 min	12 min	-	-	46 min	55 min
Stent retriever	-	EmboTrap Preset 4 × 20	-	Trevo 4/20	-	Solitaire 4x20	-	-	-	-	Trevo 3/20	Solitaire 4x40
Catheter	SOFIA5	SOFIA5 SOFIA6	SOFIA5	SOFIA5	SOFIA6	SOFIA6	SOFIA5	SOFIA5	SOFIA5	-	SOFIA5	SOFIA5
Attempts	2	5	2	1	1	1	12	1	1	-	1	3
mTICI	3	2b	3	3	2b	2b	2b	3	0	0	3	2b
Complication brain	-	Local SAH	-	-	-	Local SAH PH2 and remote hemorrhages due to i.v. thrombolysis			-	-	-	PH2
Complication neck	-	-	Cervical hematoma not requiring further therapy	Cervical hematoma not requiring further therapy	-	-	-		-	-	-	-
Onset to reperfusion	246 min	320 min	271 min	398 min	307 min	267 min	440 min	518 min	-	-	226 min	249 min

**Table 2:** Comorbidities

	%
Hypertension	83.3%
Coronary artery disease	8.3%
Peripheral artery disease	33.3%
Atrial fibrillation	66.6%
TIA/stroke before	16.7%
Congestive heart failure	16.7%
Diabetes mellitus	16.7%
Hyperlipidemia	58.3%

### Imaging

All patients presented no to minimal early ischemic stroke changes on initial CT scan with a median ASPECTS of 8, ranging from 8 to 10. Nine patients had a left-sided M1 occlusion, one patient a right-sided M1 occlusion, and 2 patients a right-sided M2 occlusion. Four patients had aortic-biiliac occlusion. The median B.A.D. score was 3 (range 2–4) for the remaining 8 patients with secondary DCP after unsuccessful cervical access via the transfemoral route.

### Time metrics and outcome

The median time from femoral to carotid puncture amounted to 30 min, ranging from 15 to 120 min. In patient 8, the DCP took place 120 min after groin puncture as the senior interventionalist was performing a simultaneous emergent aneurysm coiling at a distant campus and was not able to join the fellow’s MTE earlier.

The median procedure time was 88 min (range 13–140) with the median time from stroke onset to reperfusion 313.5 min, ranging from 246 to 518 min. The median time from DCP to reperfusion was 23 min (12–73). A successful recanalization (mTICI 2b–3, Table 1) was achieved in 10 out of 12 cases (83.3%).

In patient 9, DCP was performed uneventfully, yet MTE failed (TICI 0). Patient 10 presented with a severe bilateral aortoiliac occlusive disease. After failed catheterization attempts of the iliac arteries on both sides, bailout DCP was intended, yet unsuccessful, likely due to severe atherosclerotic plaques of the carotid artery. No complication occurred in this instance. Across all cases, we encountered no embolizations into new territories. We noted one local subarachnoid hemorrhage and 2 ECASS type II parenchymal hematomas, both in patients that had received systemic thrombolysis. The median NIHSS at discharge was 11.5, ranging from 2 to 19. In patients who survived, the median mRS counted 4 (2–4). As for the ischemic changes on the CT, the median ASPECT score at discharge was 8 (3–8). The mortality rate was 50% (*n* = 6). This included one patient with thrombolysis-associated cerebral hemorrhage, one patient with failed DCP, and one patient with failed MTE after successful DCP. Two patients died due to pneumonia and multiorgan failure. One patient died due to respiratory insufficiency and multiorgan failure.

### Procedural aspects and complications of DCP-MTE

**Fig. 1:**
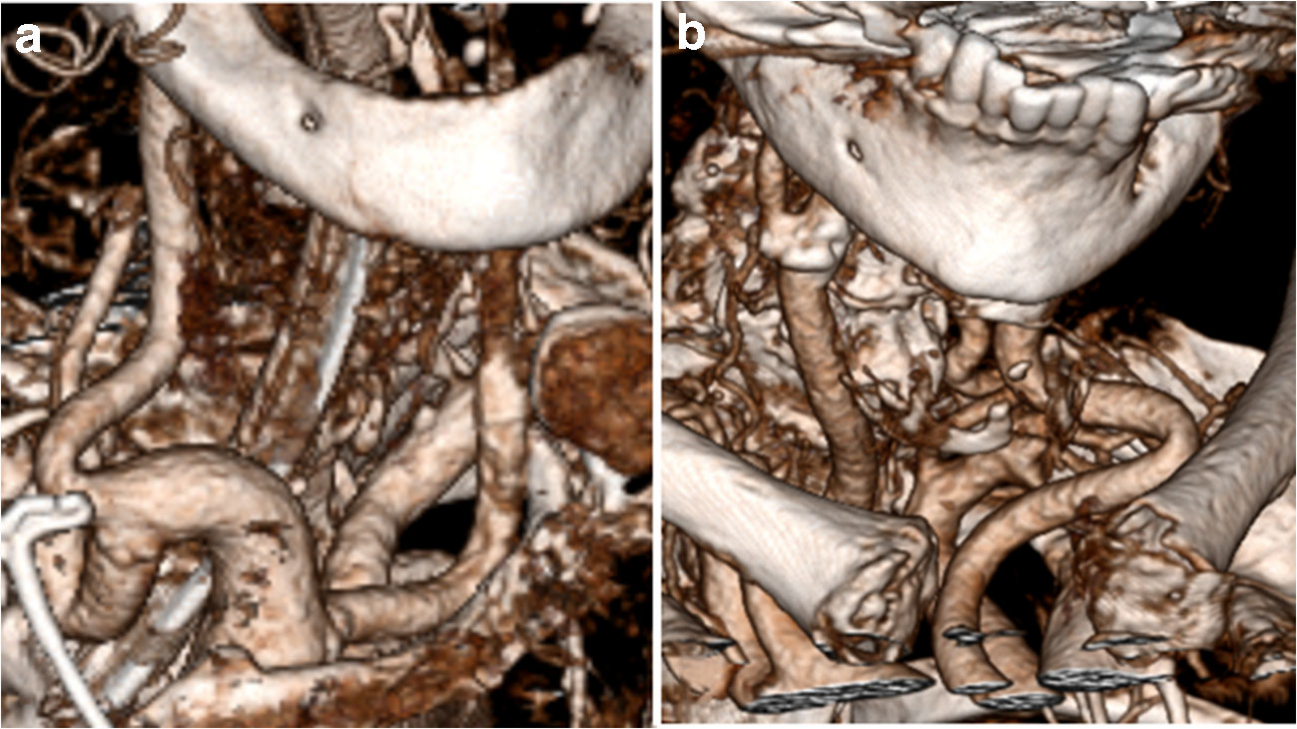
Difficult anatomy for transfemoral vessel selection requiring bail-out DCP after multiple futile catheterization attempts each. **a** Bovine arch and severe cervical vessel elongations. **b** Severely elongated and acutely angled left CCA

The standard femoral approach was attempted in 10 patients. The most common reasons for DCP were an unfavorable anatomy of the aortic arch, acute supra-aortic vessel take-offs, and carotid tortuosity (see Table 1 for details, Fig. 1). Four patients had bifemoral aortoiliac occlusive disease making a trans-femoral approach impossible. In 2 cases (patients 2 and 5), we therefore primarily used a carotid access without previous femoral puncture. In all cases, the common carotid artery (CCA) was punctured.

**Fig. 2:**
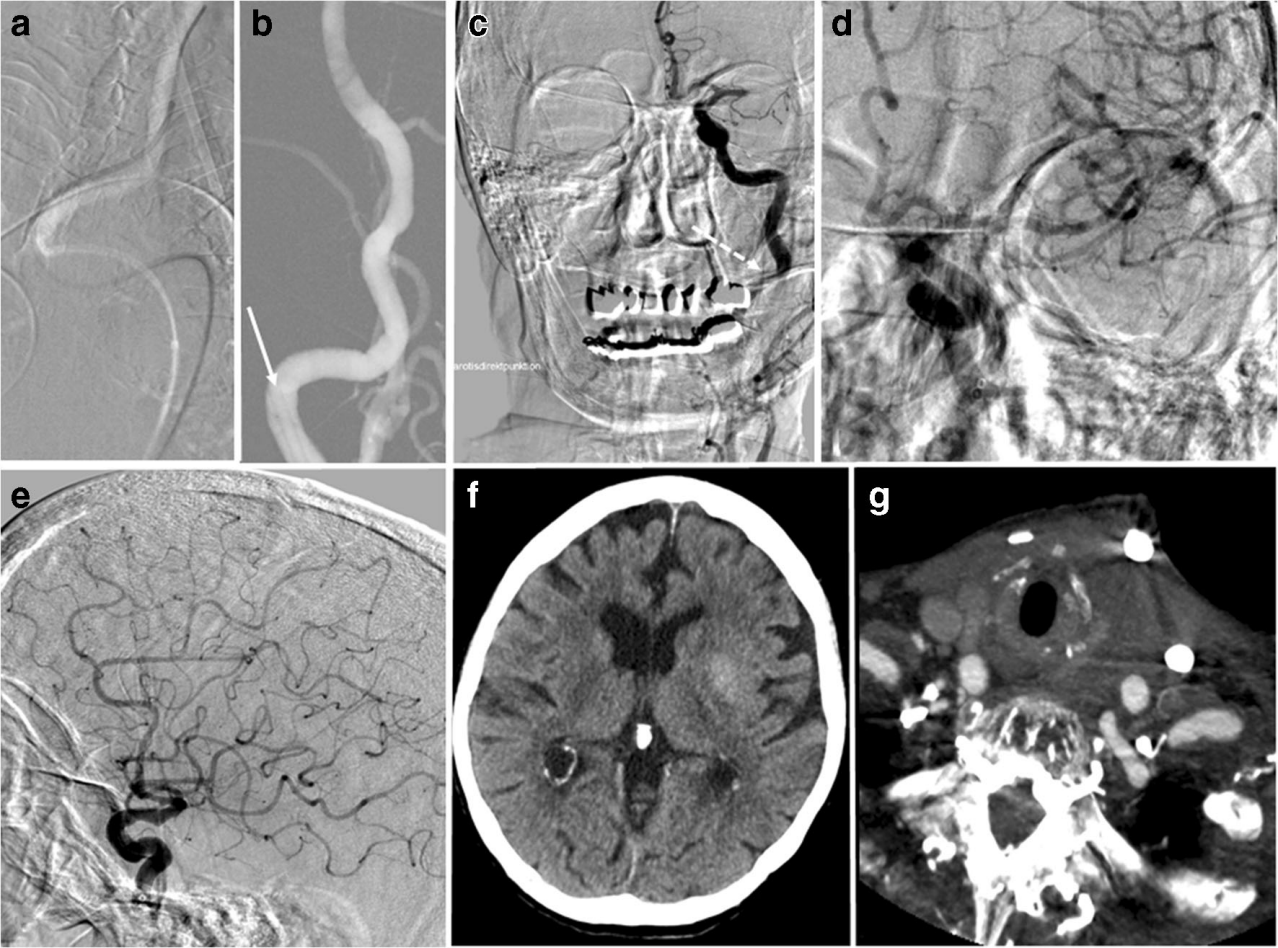
A 92-year-old female patient (no. 3) with severe iliac vessel elongation and type 3 arch (**a**). It was impossible to obtain a stable position within the left carotid for 30 min. Conversion to DCP under conscious sedation (road map via the microdilator, 6F sheath in place (**b**, arrow). Angiogram via the sheath (**c**, arrow) showing M1 occlusion. Complete recanalization after the second wADAPT pass with a 5F aspiration catheter (SOFIA) (**d**–**e**), 18 min after DCP. Follow-up after 24 h shows limited striatal infarction (**f**) and moderate regional cervical hematoma not requiring further therapy (**g**)

**Fig. 3:**
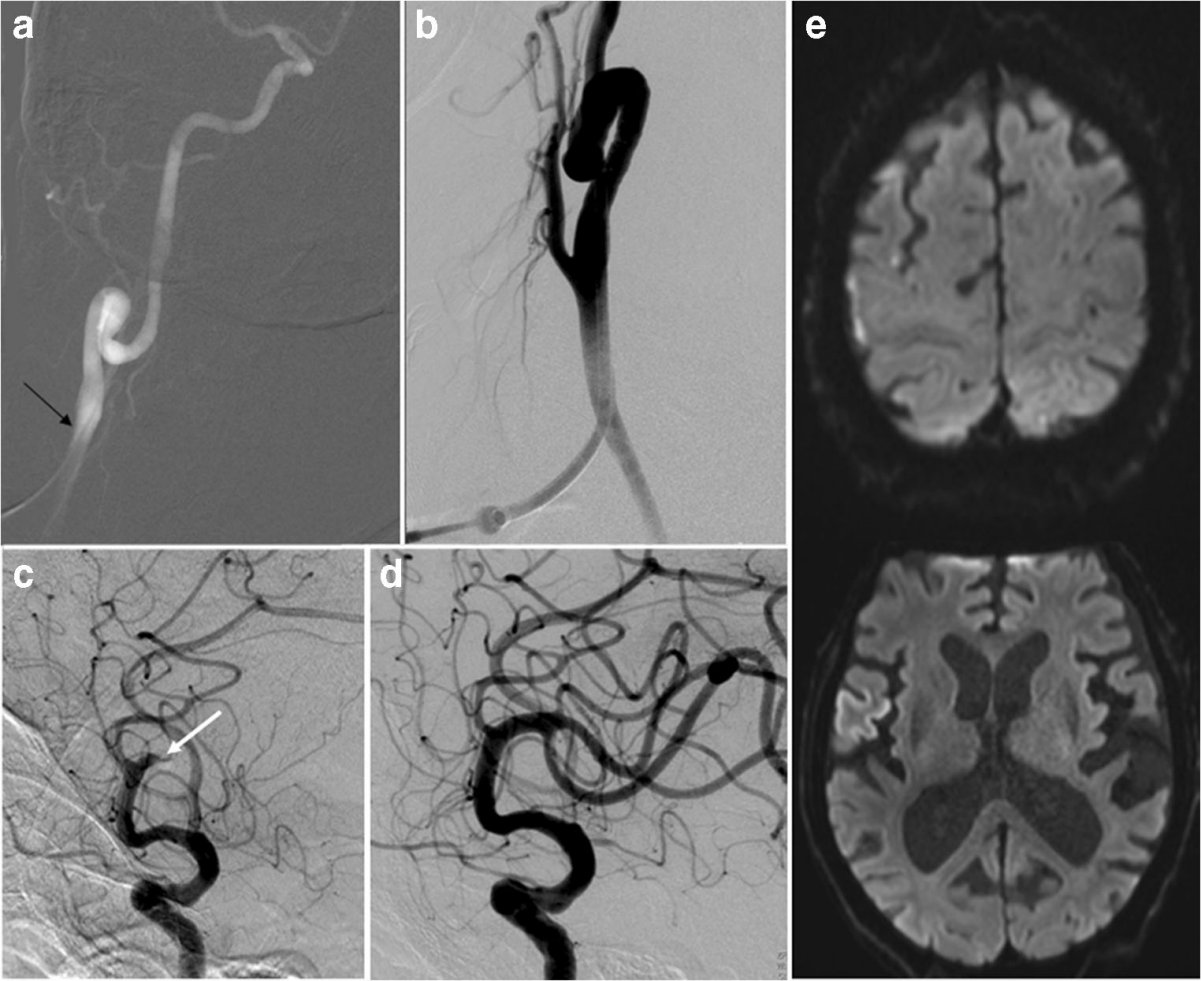
A 92-year-old male with acute occlusion of the dominant inferior M2 occlusion, NIHSS 9. MTE via secondary DCP. **a** Road map via micropuncture sheath. **b** Run via the 6F standard sheath. **c** Occclusion of the dominant inferior M2 division. **d** First pass complete recanalization 20 min after DCP. **e** Follow-up DWI next day shows limited gray matter ischemia of the opercular region and some scattered cortical ischemic foci

In 5 cases, MTE was performed using stent-retrievers combined with manual distal aspiration, and in 5 cases, a distal aspiration technique (ADAPT, wADAPT) was performed (Figs. 2 and 3); the materials used are listed in Table 1. The median number of attempts was 1.5 (range 1–12). In one case (patient 2) with a bifurcational embolus, the double stent-retriever technique was successfully performed.

For vascular closure, an AngioSeal device was used in 6 patients. In 6 cases, a manual, ultrasound-controlled compression of the puncture site was used to achieve hemostasis. Two patients developed a self-limiting cervical hematoma not requiring any further treatment.

## Discussion

In this case series, we describe a single-center experience with DCP in 12 consecutive patients undergoing MTE for acute anterior circulation ischemic stroke caused by M1 or M2 occlusions over a 2-year period. We show that primary and secondary DCP appears to be technically efficient, and fast in an otherwise challenging subset of stroke patients with 91.7% successful vascular access (11 out of 12 cases), an 83.3% overall successful recanalization rate (TICI 2b–3, 10 out of 12 cases), a median DCP to reperfusion time of 23 min, and no significant DCP-related complications.

Catheterization of the distal cervical target vessel is a critical part of the MTE procedure that may be straight forward or at least manageable in most patients, but can be cumbersome and time-consuming at times. Occasionally, however, it will be frankly impossible, even for well-trained neurointerventionalists (about 5% in one dedicated series [14]). Long catheterization times have been shown to negatively impact clinical outcome [3–5, 14]. It is thus desirable to keep the vascular access phase as short as possible. Numerous factors such as unfavorable aortic arch anatomy (type II and III aortic archs), acute angled common carotid take-offs, elongations, kinks, and loops as well as proximal CCA stenosis negatively impact on target vessel catheterization times [3–5, 14, 15]. Furthermore, left anterior circulation stroke has been shown to be a risk factor for difficult catheter access [14]. Accordingly, 7 out of our 10 patients who were primarily scheduled for transfemoral MTE had left-sided M1 occlusions. In these situations, DCP appears to be an efficient bail-out strategy. Another reason for DCP in 4 of our patients was severe aorto-biiliac occlusive disease (Leriche syndrome), unamenable to catheter passage without prior time-consuming recanalization or angioplasty procedures.

DCP is a well-described procedure in neurointervention, but has mainly been performed in the remote past representing the regular vascular access in the early days of cerebral angiography [6]. With the development of longer, more flexible and steerable catheters and wires, regular access switched to transfemoral [7, 8]. Since then, DCP has only anecdotally been described in various neurointerventions such as carotid stenting and aneurysm coiling [16–18].

Recently, there has been a renewed interest in DCP in the context of stroke MTE. Until now, however, only a few small case-series DCP for stroke MTE have been published [19–22]. In the present case series, we achieved a successful DCP in 91.7% of patients with a consecutive successful recanalization (TICI 2b–3) in 90.9% of these successful DCP cases. This is in line with the results from Roche et al. who demonstrated successful DCP in 91% out of 11 treated patients with a recanalization rate of 73%. Jadhav et al. achieved a recanalization rate of 87.5% out of 7 treated patients [20]. Neck hematoma appears to be the most common complication following a DCP with an incidence ranging from 9 to 14% in these series [19–21]. Roche et al. report one neck hematoma not requiring further treatment. Furthermore, Roche et al. report an ICA dissection in one patient. We observed no severe complications related to DCP with a neck hematoma prevalence of 16.7%; however, none required further therapy.

Neck hematoma is a potentially severe complication which may lead to airway compression and may therefore require immediate surgical evaluation. Therefore, a meticulous puncture technique with a micropuncture set, ultrasound guidance, and reliable hemostasis after the intervention are crucial. The latter can be achieved by manual compression, ideally aided with the ultrasound transducer which provides an even squared compression area with the major advantage of accurately ensuring vascular patency thereby limiting the possibility of intraluminal thrombus formation as well as exclusion of potential relevant cervical hematoma development.

So far, no closure devices have been designed specifically for the carotid artery, although the successful off-label use of existing closure devices such as the AngioSeal has been reported by others and was safely performed in 6 patients in our series [23]. Plug dislodgment or local thrombus formation with subsequent cerebral embolism remains concerns that must not be neglected, and safety and efficacy of such devices in DCP should be addressed in larger series.

The type of postoperative hemostasis was dependent on the treating physician’s discretion. Generally, an argument for us to use the closure system was prior bridging lysis, while cervical cachexia with little subcutaneous fat to accommodate the superficial plug component was considered a counter argument. Another well-established and safe option is surgical closure of the carotid puncture site, which requires the institutional 24/7 availability of a vascular surgery team.

After gaining some experience, we stopped intubating cooperative patients for the DCP. In total, 8 DCP-MTE were performed under general anesthesia and 4 under conscious sedation. In these four cases, the procedure was well tolerated. We think that patient information, if neurologically possible, and sufficient, preferentially ultrasound-guided local anesthetic infiltration down to the perivascular connective tissue are helpful to avoid patient discomfort and ensure a smooth procedure. We do not suggest to do direct punctures in uncooperative awake patients.

Both road-map (in case of secondary DCP) and ultrasound guidance are valid options for safe and accurate carotid puncture. We prefer the latter, because the puncture site can be evaluated for potential atheroma, and the micropuncture process can be visualized to avoid dissection and puncture of the jugular vein or an enlarged thyroid that are potentially located in the needle trajectory. Furthermore, radiation exposure aspects favor ultrasound guidance. A surgical cut-down of the common carotid artery in the angiosuite has also been described for MTE [24, 25]; however, this is a relatively invasive and likely rather time-consuming method as compared to an ultrasound-guided carotid puncture.

The transradial approach has been extensively investigated in the interventional cardiology field and has been shown to be superior to the transfemoral route concerning safety aspects and patient satisfaction [26]. Gradually, it is also being adopted by the neurointerventional community with compelling results for both diagnostic and therapeutic procedures reported in the recent literature [27]. Several case series have shown the suitability of the transradial access for MTE with both safe and effective recanalization of anterior and posterior circulation vessel occlusions [13, 28–30]. This approach most likely represents the safer alternative to DCP and seems to be the preferable bail-out access method for many unsuccessful transfemoral cases, if not used upfront already. On the other hand, there are hostile aortic arches with severe adjacent supraaortic vessel tortuosity that do impose significant challenges for both transfemoral and transradial routes. In these situations, familiarity with DCP may be particularly valuable. We feel that another potential advantage of the DCP approach may be based on the relative proximity of the vascular access point to the target occlusion with a relatively short distance to the thrombus and less intervening curvatures resulting in excellent transmission of torque and forces thus enabling straightforward and efficient MTE in otherwise cumbersome cases.

For DCP, no specific devices such as arterial sheaths or catheters are available so far (aside from the TCAR procedure materials [Silk Road Medical, Sunnyvale, CA] which involves a direct surgical cut-down of the common carotid artery). One of our operators prefers long 6F sheaths (90 cm Destination, Terumo, Tokyo, Japan) which can be connected to a standard hemostatic valve/y-connector. Furthermore, the working position and telescoping of catheters are similar to the standard femoral technique and may also be advantageous in terms of operator radiation exposure. The drawback of this approach may be difficulties in introducing the long sheath in the carotid artery due to its relatively longer and stiffer dilator which can cause significant vessel displacement and may lead to mural vessel damage. On the contrary, when using a short vascular sheath, which cannot be connected to a y-connector, the insertion of a floppy aspiration catheter through the sheath valve can be a bit more challenging. It can be achieved by insertion of a standard hydrophilic guide wire into the intermediate which is then pushed to open the valve after placing the distal end of the intermediate catheter directly on the valve. With the valve thus opened, the aspiration catheter can be tracked along without difficulty. In case of removal of a large thrombus that cannot be ingested into the intermediate catheter, prolonged and forceful manual aspiration on the carotid sheath is of utmost importance to prevent re-embolization. In such situations, thrombus material may get stuck in the most proximal part of the sheath underneath the valve. Thrombus removal may then require manually opening of the sheath valve leaflets which can be accomplished by insertion of a thin instrument such as an introducer. Only after complete removal of all thrombus should a control angiogram be performed.

This series is limited by the small case number as well as the retrospective design with its known inherent limitations. Representing a bailout vascular access strategy for multimorbid patients, the clinical outcome in this patient cohort was only moderate to poor (50% mortality). Obvious potential reasons are advanced age (median 85.5 years), a high rate of serious comorbidities, and a pre-existing functional impairment prior to the incident stroke (66.6% of patients had a pre-stroke mRS > 2). Although no definitive conclusions can thus be drawn from this small series, it is nevertheless fair to state that none of the poor outcomes observed had any plausible relation to the DCP. In the future, predictive CTA parameters on cervical navigability via the transfemoral route should be evaluated, in order to directly proceed with DCP in selected cases. Although we would consider treating also distal internal carotid artery occlusion with DCP-MTE, we did not encounter this specific situation and thus cannot give practical details. Furthermore, potential safety issues of the procedure cannot be eliminated definitively in the light of this and other published series of moderate size only.

Since we established the transradial route in our department substantially later than DCP in the final year of the investigated time span, our results are skewed towards DCP as alternative access approach. Potentially, a part of our cases could have also been done by transradial MTE.

## Conclusion

This case series suggests that DCP is an efficient and fast bailout vascular access technique for MTE in patients with anterior circulation ischemic stroke in whom treatment would otherwise be very difficult or even impossible. We describe high recanalization rates comparable to the transfemoral approach for both M1 and M2 occlusions, and we did not encounter any severe puncture-associated complications. Further investigation of this approach for highly selected stroke patient cohorts seems warranted.

## Data Availability

The datasets used and/or analyzed during the current study are available from the corresponding author on reasonable request.
